# IFABP levels predict visceral malperfusion in the first hours after open thoracoabdominal aortic repair

**DOI:** 10.3389/fcvm.2023.1200967

**Published:** 2023-06-27

**Authors:** Panagiotis Doukas, Cathryn Bassett, Hanif Krabbe, Jelle Frankort, Michael J. Jacobs, Moustafa Elfeky, Alexander Gombert

**Affiliations:** Department of Vascular and Endovascular Surgery, University Hospital Aachen, RWTH Aachen University, Aachen, Germany

**Keywords:** intestinal ischemia, visceral malperfusion, thoracoabdominal aorta, open aortic repair, postoperative management, aortic aneurysm

## Abstract

**Introduction:**

Intestinal ischemia after open thoracoabdominal aortic repairs, is a rare but devastating complication, associated with high mortality. Notoriously challenging to diagnose, visceral malperfusion necessitates immediate surgical attention. Intestinal fatty acid-binding protein (IFABP) has been proposed as a biomarker for the diagnosis of intestinal wall damage. In this prospectively conducted, observational study we evaluated the diagnostic capacity of IFABP levels in patients' serum and their correlation with visceral malperfusion.

**Methods:**

23 patients undergoing open thoracoabdominal aortic repairs were included in this study and 8 of them were diagnosed postoperatively with visceral malperfusion—defined as a partial or complete thrombotic occlusion of the superior mesenteric artery and/or the coeliac trunk. IFABP levels and laboratory parameters often associated with intestinal ischemia (leucocytes, CRP, PCT and lactate) were measured at baseline, directly postoperatively, and at 12, 24 and 48 h after surgery. Postoperative visceral malperfusion—as revealed in CT angiography—was assessed and the predictive ability of IFABP levels to detect visceral malperfusion was evaluated with receiver-operator curve analysis.

**Results:**

Patients with visceral malperfusion had a relevant risk for a fatal outcome (*p* = .001). IFABP levels were significantly elevated directly postoperatively and at 12 h after surgery in cases of visceral malperfusion. High IFABP concentrations in serum detected visceral malperfusion accurately during the first 12 h after surgery, with the maximum diagnostic ability achieved immediately after surgery (AUC 1, Sensitivity 100%, Specificity 100%, *p* < .001).

**Conclusion:**

We conclude, that IFABP measurements during the first postoperative hours after open thoracoabdominal aortic surgery can be a valuable tool for reliable and timely detection of visceral malperfusion.

## 1. Introduction

Open surgical treatment of thoracoabdominal aortic aneurysms (TAAA) often requires reconstruction of the viscerorenal aortic segment, alongside with aortic cross-clamping and exposure to extracorporeal circulation, which may impair the integrity of the intestinal wall (1). With an incidence of 3% (2) to 9% (3) visceral malperfusion and intestinal ischemia are relatively rare, but devastating complications in fields of TAAA surgery, associated with high mortality rates [50%–90% (4)]. Furthermore, these cases are often accompanied by systemic inflammatory response and sepsis, as well as intestinal necrosis requiring surgical treatment. However, fluctuations in patient's fluid homeostasis, cardiopulmonal instability and deep sedation during the first postoperative hours may mask clinical and laboratory symptoms and signs of visceral malperfusion and hinder clinical suspicion and diagnosis.

A promising biomarker strongly associated with the incidence of bowel ischemia is intestinal fatty acid-binding protein (IFABP) (5). This 15 kDa protein is expressed in the cytoplasm of mature enterocytes found at the end of the intestinal villi (6). This area of the gut wall is the most distant from the intestinal capillary network and, as such, it bears the highest risk to be the first to succumb to malperfusion (7). Under the premise of this mechanism, many studies have investigated the clinical applicability and reliability of IFABP for the diagnosis of intestinal necrosis (1, 5, 6). The focus of its clinical relevance is placed on the detection of mesenteric ischemia affecting the small intestine, since the concentration of the protein is in this segment twenty times higher than in the large intestine (8). In the settings of open abdominal and thoracoabdominal aortic surgery, we demonstrated that elevation of IFABP after surgery and on the first postoperative day could reliably detect intestinal necrosis (1).

Expanding on the findings of this previous work, in this prospectively conducted study we investigated the dynamic changes of IFABP levels in patients' serum during the first postoperative hours after open TAAA repair and its correlation with the diagnosis of visceral malperfusion.

## 2. Patients and methods

### 2.1. Study design

In this observational study conducted at a single center, 23 patients who underwent open TAAA reconstructions between January 2019 and February 2023 were included. The study protocol was reviewed and approved by the ethic committee of the University Hospital Aachen (EK010/19) and designed according to the Declaration of Helsinki and the STROBE criteria. The apriori study protocol describing material acquisition was registered at clinicaltrials.gov/ct2/show/NCT04087161. Patients provided written informed consent before participating in the study. Pregnant women and those under 18 years of age were excluded. All included cases were elective, with patent coeliac trunk and superior mesenteric arteries at the time of the operation. Digital medical records and clinical charts were used to collect data on patients' medical history and demographic details.

### 2.2. Surgery

The surgical procedure for TAAA reconstructions, including the reconstruction of the visceral aortic segment, has been previously described in the literature (9). Exposure of the thoracoabdominal aorta was achieved through thoracolaparotomy. The abdominal aorta was approached transperitoneally. In all included cases a femoral-femoral cannulation via the left femoral vessels was applied. After the proximal aortic clamp was placed, the extracorporeal circulation was initiated to provide distal aortic perfusion. The time interval starting from the placement of the proximal aortic clamp until its removal at the end of the reconstruction, is later referred to as “cross-clamping time”. During the reconstruction of the visceral aortic segment, selective perfusion of all visceral arteries was achieved via extracorporeal circulation, enabling a flow of at least 500 ml/min per catheter. CUSTODIOL® solution (HTK, Dr. Franz Köhler Chemie GmbH, Bensheim, Germany) was infused into both kidney arteries, and the mean arterial pressure was kept at 90 mmHg or higher throughout the procedure. Mild systemic hypothermia (32°C–33°C), cerebrospinal fluid drainage and intraoperative monitoring of motor-evoked potentials were implemented as protective measures to reduce the risk of spinal cord ischemia and postoperative neurologic deficits.

### 2.3. Measurements

To measure IFABP concentrations in serum, blood samples were collected from patients at baseline, immediately after surgery, and at 12, 24, and 48 h postoperatively. The samples were stored at −80°C after centrifugation, and IFABP concentrations were calculated using ELISA (RayBio® Human FABP2 ELISA Kit, RayBiotech, Norcross, USA) according to the manufacturer's recommendations. After all reagents and samples were prepared, 100 μl of sample were added in each well and were incubated for 2.5 h at room temperature. Then, 100 μl of the prepared biotin antibody were added to each well and were allowed to incubate for 1 h, before 100 μl Streptavidin solution were added. After further 45 min of incubation at room temperature, 100 μl of TMB One-Step Substrate Reagent were added. After 30 min of incubation at room temperature, the reaction was arrested with 50 μl of Stop Solution and the microplate was immediately read at 450 nm with the Tecan Spark 10 M Luminescence Multi Mode Microplate Reader (Tecan, Männedorf, Switzerland). The intra-assay coefficient of variation was 10%, and the inter-assay coefficient was 12%. The normal values for circulating IFABP in healthy, non-obese population is 4.8 ng/ml [Interquartile range 3.7–8] (10).

Routine laboratory parameters, including leucocyte count, levels of procalcitonin (PCT) and C-reaction protein (CRP) were obtained at baseline and at 12, 24 and 48 h postoperatively. Lactate was measured in arterial blood collected in a heparinized plastic syringe. The collected sample was immediately analyzed in the blood gas analyzer ABL90 Flex (Radiometer, Brønshøj, Denmark).

### 2.4. Endpoints

The primary endpoint and focus of this study was postoperative visceral malperfusion and its correlation with IFABP levels in serum. Patients with reduced arterial flow to mesenterium, because of acute partial or complete thrombotic occlusion of the superior mesenteric artery and/or coeliac trunk, as revealed by angiographic scan, were diagnosed with visceral malperfusion (11). In cases of only partial occlusion of the visceral arteries, patients with moderate or high grade stenosis were included in the “visceral malperfusion” group. Examples of CT-angiograms of included patients with partial and complete occlusions of the visceral arteries are presented in Supplementary Figure S1. The diagnosis of visceral malperfusion was based solely on angiographic findings in the computer tomography scan, regardless of laboratory parameters and clinical presentation. The patients with clinical suspicion of intestinal ischemia, elevated serum lactate and angiographic confirmation of visceral malperfusion required surgical revision.

Secondary endpoints were the associations of visceral malperfusion and adverse events during the postoperative phase. The diagnostic criteria for multi-organ dysfunction syndrome (MODS) was fulfilled when two or more vital organ systems failed (12). Sepsis was diagnosed in patients with active infection and an increase of their daily Sequential Organ Failure Assessment (SOFA)-Score by 2 points or more in comparison to the previous day (13). According to the Berlin definition, the diagnosis of acute respiratory distress syndrome (ARDS) was applied (14). Patients without a history of liver disease and a spontaneous international normalized ratio >1.5 accompanied with acute onset of jaundice were diagnosed with acute liver injury (15). Renal replacement therapy (RRT) was initiated in cases of severe metabolic acidosis and hyperkaliaemia, anuria and refractory volume overload (16).

### 2.5. Statistics

The absolute frequencies and percentages of the total sample are used to report categorical variables, while mean (±standard deviation) is used to present continuous variables. In the results, significance levels are indicated by (*) for *p* < .05, (**) for *p* < .01, and (**) for *p* < .001, with a 95% confidence interval (CI). Correlations of visceral malperfusion and postoperative complications and patient demographics were tested using univariable, logistic regression. IFABP serum levels were logarithmically transformed to achieve a normal distribution and one-way ANOVA was used to test for correlations between IFABP serum levels and the onset of visceral malperfusion. The diagnostic capacity of serum lactate and IFABP levels was evaluated using receiver-operating-characteristics (ROC) analysis and the optimal cut-offs were obtained with the Youden-Index. Data analysis was performed using the SPSS software (SPSS Inc., Chicago IL) and graphics were created with the GraphPad Prism version 8.0.0 for Windows (GraphPad Software, San Diego, California USA).

## 3. Results

There were 23 patients (*n* = 20 men) included in this study with a mean age of 51.5 ± 11.7 years. 8 of these patients were diagnosed with visceral malperfusion during the postoperative phase. The details of the patients' comorbidities are displayed in Table 1. The onset of visceral malperfusion was not associated with any comorbidities and it was independent from both aortic cross-clamping time and duration of surgery, although we observed a statistically insignificant trend for longer procedures in the visceral malperfusion group (535 ± 92.5 min vs. 490 ± 103.1 min, *p* = .21). The most common reconstruction was type II repairs (Crawford classification). Postoperatively, patients with visceral malperfusion required in 50% of the cases a re-laparotomy for surgical revision of the visceral bypasses. The 4 patients in the visceral malperfusion group, that were not surgically revised presented partial thrombosis of the SMA. Two of these cases were treated conservatively with therapeutic anticoagulation and the other two succumbed to circulatory arrest before surgical revision. Resection of intestinal segments was necessary in 3 cases (38%). MODS and acute liver injury were significantly more common in these patients (MODS: 63% vs. 5%, *p* < .001; acute liver injury: 50% vs. 5%, *p* = .002). We also observed a trend for the onset of sepsis but without statistical significance (Sepsis: 63% vs. 32%, *p* = .14).

**Table 1:** Patient demographics and postoperative details.

	No visceral malperfusion (*n* = 15)	Visceral malperfusion (*n* = 8)	*p*-value
Demographics			
Age (years)	51.5 ± 11.7	48.8 ± 16.4	.95
Men	16 (73)	4 (50)	.26
Obesity	2 (9)	1 (13)	.79
Body mass index (kg/m^2^)	23.4 ± 2.4	26.3 ± 6.2	.46
Smoking	7 (32)	2 (25)	.73
Chronic obstructive pulmonary disease	5 (23)	2 (25)	.9
Hypertension	15 (68)	8 (100)	.1
Chronic kidney disease (eGFR <60 ml/min/1.73 m^2^)	10 (46)	5 (63)	.43
Type of TAAA			.7
I	1 (5)	2 (25)	
II	10 (46)	2 (25)	
III	5 (23)	2 (25)	
IV	5 (23)	1 (13)	
V	1 (5)	1 (13)	
Duration of surgery (minutes)	490 ± 103.1	535 ± 92.5	.21
Cardiopulmonary bypass time (minutes)	141.5 ± 51.1	156.62 ± 37.7	.52
Postoperative data			
30-day-mortality	0 (0)	3 (38)	.1
Intestinal resection	0 (0)	3 (38)	.002**
Surgical revision of the visceral bypass	0 (0)	4 (50)	<.001***
Intensive care unit stay (days)	30.2 ± 31.6	41.3 ± 38.4	.2
Hospital stay (days)	43.7 ± 27.4	64.1 ± 47.1	.42
Pneumonia	13 (59)	7 (88)	.2
Acute respiratory distress syndrome	10 (46)	5 (63)	.43
Sepsis	7 (32)	5 (63)	.14
Multi-organ dysfunction syndrome	1 (5)	5 (63)	<.001***
Acute liver injury	1 (5)	4 (50)	.002**
Renal replacement therapy	12 (55)	7 (88)	.1
Apoplex	4 (18)	1 (13)	.72
Delirium	9 (41)	2 (25)	.44

TAAA, thoracoabdominal aortic aneurysm; eGFR, estimated glomerular filtration rate.

** for *p* <.01 and *** for *p* <.001.

**Figure 1:**
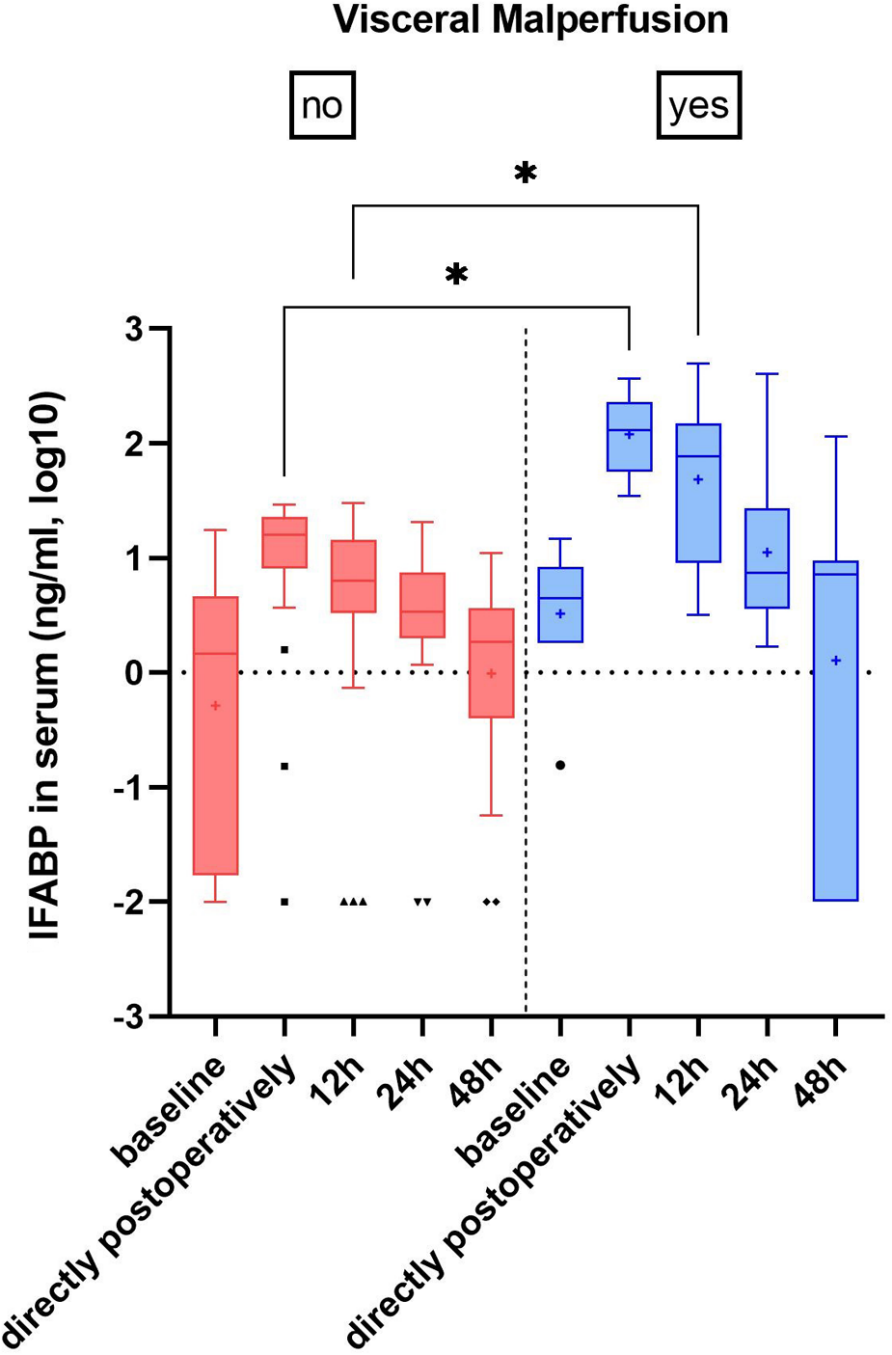
IFABP levels in serum.

IFABP serum levels were postoperatively elevated in all patients, however at admission in the ICU the increase was ten-fold in patients with visceral malperfusion (154.4 ± 11.4 ng/ml vs. 14.9 ± 8.7 ng/ml, *p* = .02) and persisted for the first 12 h (125.6 ± 163.8 vs. 9.2 ± 8.4, *p* = .01) (Figure 1). Among these patients, we found a trend for higher IFABP levels in cases that necessitated intestinal resection, however without statistical significance (IFABP at 12 h: 224.9 ± 247 vs. 66.1 ± 67.8; *p* = .21) (Supplementary Table S1; Supplementary Figure S2). We observed a dynamic decrease in the subsequent time points. Serum lactate, C-reactive protein (CRP), procalcitonin (PCT) and leucocytes—all markers routinely examined in case of suspicion of visceral malperfusion—displayed only a trend of elevation during the early postoperative phase, without reaching statistical significance (Table 2).

**Table 2:** Dynamics of leucocytes, CRP, PCT, lactate and IFABP in serum.

	No visceral malperfusion (*n* = 15)					Visceral malperfusion (*n* = 8)				
	Baseline	Admission ICU	12 h	24 h	72 h	Baseline	Admission ICU	12 h	24 h	72 h
Leucocytes (/nl)	7.3 ± 3.2	-	9.1 ± 3.5	10.7 ± 3.6	10.9 ± 3.7	9.5 ± 3.4	-	10.6 ± 2.2	11.7 ± 3.3	12.4 ± 4.1
CRP (mg/L)	23.3 ± 66.1	-	93.5 ± 47.8	240.4 ± 49.2	260.6 ± 62.5	56.3 ± 108.3	-	93.8 ± 69.1	212.8 ± 122.8	215.3 ± 85.4
PCT (ng/ml)	-	-	12.4 ± 12.7	13.9 ± 14.1	10.6 ± 11.4	-	-	32.5 ± 41.4	23.3 ± 37.8	25.3 ± 36.7
Lactate (mmol/L)	.9 ± 1.2	3.2 ± 4.4	2.3 ± 1.8	1.6 ± 1.1	1.1 ± .5	.6 ± .2	4.2 ± 3.9	5.5 ± 5.5	5 ± 7.7	2.4 ± 2.5
IFABP (ng/ml)	2.8 ± 4	14.9 ± 8.7	9.2 ± 8.4	4.9 ± 4.5	2.3 ± 2.4	5 ± 4.9	154.4 ± 111.4	125.6 ± 163.8	59.7 ± 139.3	17.6 ± 39.2

CRP, C-reactive protein; PCT, procalcitonin; IFABP, intestinal-fatty acid binding protein.

ROC-Curve analysis for IFABP levels postoperatively revealed high diagnostic accuracy for visceral malperfusion during the first 12 h. The maximum accuracy was reached at patient's admission on ICU (AUC = 1, Sensitivity 100%, Specificity 100%, *p* < .001 for a cut-off of 32 ng/ml). Persistent elevation of IFABP levels 12 h postoperatively could still accurately predict visceral malperfusion (AUC = .835, Sensitivity 95.5%, Specificity 75%, *p* = .006 for a cut-off value of 21.7 ng/ml). In comparison, the clinically established lactate levels in serum did not reach an equivalent diagnostic capacity postoperatively with a maximum AUC of.784 (Sensitivity 87.5%, Specificity 60%, *p* = .02) at 12 and 24 h (Table 3).

**Table 3:** ROC-curve analysis for IFABP and lactate in serum.

	IFABP					Lactate				
	AUC	*p*-value	Cut-off	Sensitivity (%)	Specificity (%)	AUC	*p*-value	Cut-off	Sensitivity (%)	Specificity (%)
Baseline	.636	.26	4.23	72.7	50	.585	.48	.55	50	63.6
Admission ICU	1	<.001	32	100	100	.6	.4	2.15	75	50
12 h	.835	.006	21.7	90.1	75	.784	.02	2.05	87.5	60
24 h	.733	.05	11.2	95.5	50	.784	.02	1.5	87.5	65
48 h	.54	.74	6	95.5	50	.71	.09	1.3	62.5	70

## 4. Discussion

### 4.1. Dynamic of IFABP levels

IFABP levels did not differ between the two groups at baseline. Directly after surgery IFABP was elevated in both groups—albeit tenfold higher in the visceral malperfusion group—and declined in all subsequent time points. This dynamic can be understood through the physiologic expression of IFABP in mature enterocytes, which reside at the tip of the intestinal villi and are the first to succumb under ischemic conditions, leaking IFABP in circulation (17). The reasons for the ischemic injury of the gut wall are multifactorial and although surgical techniques—like distal aortic perfusion and selective visceral perfusion—have been optimized to safeguard mesenterial oxygen supply during reconstruction of the viscerorenal segment, enterocyte damage cannot be eliminated altogether.

Firstly, aortic-cross clamping, opening the aneurysm sack and connection of the selective, visceral perfusion cannulas to provide volume-controlled blood flow to the intestine may cause a short period of absolute ischemia of the gut wall (1). Although, the time of this maneuver is sought to be brought to a minimum, it can still cause damage to the integrity of the gut wall. The selective perfusion of the visceral vessels as well as the maintenance of distal aortic perfusion deliver protection against intestinal ischemic injury (18). Yet, insults of the microcirculatory network are still possible (19) and some authors argue that selective visceral perfusion on its own does not provide adequate oxygenation of the bowel during the reconstruction of the viscerorenal aortic segment (18, 20, 21). In fact a point of discussion is the “unnatural” laminar flow generated from the extracorporeal circulation, which may limit the mucosal microcirculation (22) and disadvantage the microvascular perfusion (23). The implementation of pulsatile flow in the extracorporeal circulation circuit in the setting of cardiac surgery, may arguably preserve microcirculation and endorgan integrity (22), however some authors argue that pulsatile flow offers no significant advantage concerning organ perfusion or inflammatory response (24). Currently, in the field of selective visceral perfusion during open TAAA repairs, the implementation of pulsatile flow has not found widespread applicability. All patients included in this study received both selective visceral perfusion and distal aortic perfusion as per protocol with a laminar flow pattern and we did not observe a significant correlation of CBP time and the onset of visceral ischemia (141.5 ± 51.1 min vs. 156.62 ± 37.7 min, *p* = .52), supporting the notion that the protective measures provided sufficient oxygenation to the intestine during aortic reconstruction.

However, tissue injury of the gut wall suffers a second hit during reperfusion after completion of the reconstruction and re-initiation of pulsatile blood flow (25). In a previous work of our group, we described that clinical presentation of ischemia-reperfusion-injury of the intestine may greatly vary from patient to patient and is a dynamic event with both local and systemic consequences (26). Thus, the secretion of IFABP in the blood stream peaks at the end of the surgery, as the aftermath of the necrosis of the mature enterocytes of at the tip of the villi, although the vitality of the intestine seems macroscopically uncompromised.

These observations are in accordance with the previous study on IFABP in open TAAA repair patients, which described a significant elevation of IFABP during and after extra-corporeal circulation (1). IFABP is cleared through the kidneys and its levels physiologically decline in the early postoperative phase (27).

### 4.2. Clinical significance of IFABP serum levels

IFABP has gained the attention of researchers as a minimal invasive tool for the diagnostic algorithm of intestinal ischemia, which can be clinically easily missed (28). Sun et al. report in their meta-analysis a pooled sensitivity of 80% and pooled specificity of 85% for detecting acute mesenterial ischemia (28). However, they also report relevant heterogeneity of the included patients in their study, which may limit the interpretation of the results. Among the included studies, both vascular and non-vascular types of acute intestinal ischemia were included, which might have influenced the heterogeneity of the study cohort. Nuzzo et al. found IFABP not applicable for detecting acute mesenteric ischemia in their cross-sectional study for both arterial and venous intestinal infarction at the time of the admission in the emergency department (6). They report higher IFABP levels in cases of late phase intestinal necrosis, but without statistical significance. This finding is in line with the observations of Schellekens et al., who also did not find significant alterations of IFABP serum concentration between mucosal and transmural bowel ischemia (29).

Although Nuzzo et al. selected their patient cohort carefully and excluded patients with bowel strangulation, the clinical manifestation of acute mesenteric ischemia in an emergency setting may still account for high variability and confounding. The patient cohort presented in our study is homogenous, treated by the same surgeon according to a standardized operative protocol and similar pathophysiologic mechanisms for the onset of visceral malperfusion. The reported 100% sensitivity and 100% specificity for high IFABP levels directly postoperatively and significant diagnostic accuracy up to the 12 h postoperatively (AUC.835, Sensitivity 90%, Specificity 75%, *p* = .006) indicate the relevance of IFABP serum levels as an adjutant to the detection of visceral malperfusion and may warrant further clinical and radiographic evaluation. We speculate that the increase of routinely monitored IFABP levels during the first hours after surgery may justify an early CT-angiogram to confirm the diagnosis of visceral malperfusion and plan the potential revision without delay.

This study is limited mainly through the small number of included patients. Open TAAA repairs are relatively rare procedures in the current era of endovascular surgery and visceral malperfusion itself is a rare complication. However, the observed differences of the IFABP dynamic between the two groups were statistically highly significant and therefore reliable. Moreover, although our surgical protocol included selective perfusion of the visceral arteries as a measure to minimize the ischemic time of the viscera, there was a small interval of absolute ischemia starting from the clamping of the viscerorenal segment until the identification and cannulation of the visceral ostia. We did not include this time interval in our study protocol, since it was kept under 5 min in all presented cases. A further weak point of this project is the heterogeneity of the investigated endpoint “visceral malperfusion”: in some patients, this would mean a partial thrombotic occlusion of the mesenteric circulation and in other cases complete transmural necrosis with necessary resection of intestinal segments. Yet, the pathomechanisms of the reported visceral malperfusion underlie the similar principles and necessitate thorough clinical evaluation, making the measurements of IFABP in patients' serum a valuable tool in the arsenal of both the vascular surgeon and the intensivist.

## 5. Conclusion

In conclusion, IFABP serum levels could reliably and accurately predict visceral malperfusion in patients after open TAAA repair. Visceral malperfusion may signify impending intestinal necrosis and elevated serum concentrations of IFABP during the first 12 h postoperatively could be an alarm sign for further clinical and radiographic evaluation of the patients at risk.

## Data Availability

The raw data supporting the conclusions of this article will be made available by the authors, without undue reservation.
