# Supplementary material for: IFABP levels predict visceral malperfusion in the first hours after open thoracoabdominal aortic repair
Source: Front Cardiovasc Med. 2023 Jun 27;10:1200967. doi: 10.3389/fcvm.2023.1200967 (PMC10333487; doi:10.3389/fcvm.2023.1200967)
Supplement: Supplementary file 1 [file Table1.docx]

Supplementary Material

**IFABP levels predict visceral malperfusion in the first hours after open thoracoabdominal aortic repair**

**Panagiotis Doukas, MD^1*^ – Cathryn Bassett^1^ – Hanif Krabbe, MD^1^ – Jelle Frankort^1^ - Michael J. Jacobs, MD, PhD^1^ – Moustafa Elfeky^1^ – Alexander Gombert, MD, PhD^1^**

^1^Department of Vascular and Endovascular Surgery, University Hospital Aachen, RWTH Aachen University, Germany

*** Correspondence:**Panagiotis Doukas, MD; Pauwelsstrasse 30, 52074 Aachen, Tel: +49 (0) 241 80-80832, E-Mail: [pdoukas@ukaachen.de](mailto:pdoukas@ukaachen.de)

Supplementary Table 1: Subgroup analysis of IFABP levels in serum (ng/mL) in the visceral malperfusion group

|  | Intestinal Resection (n=3) | No Intestinal Resection (n=5) | p-value | Surgical revision of the visceral bypass (n=4) | No Surgical revision of the visceral bypass (n=4) | p-value |
| --- | --- | --- | --- | --- | --- | --- |
| baseline | 7.7 ± 7.4 | 3.4 ± 2.5 | 1 | 6.8 ± 5.4 | 2.1 ± 2.2 | 1 |
| Admission ICU | 201.7 ± 166.5 | 126.1 ± 71.9 | .87 | 199.9 ± 117.8 | 78.6 ± 43.4 | .55 |
| 12h | 224.9 ± 247 | 66.1 ± 67.8 | .21 | 143.2 ± 207 | 96.4 ± 78.4 | .98 |
| 24h | 139.9 ± 228.2 | 11.5 ± 13.1 | .42 | 87.5 ± 176.7 | 13.4 ± 16.9 | .9 |
| 48h | 41.2 ± 63.3 | 3.4 ± 4.3 | .99 | 26.3 ± 49.3 | 3 ± 5.1 | .99 |

**
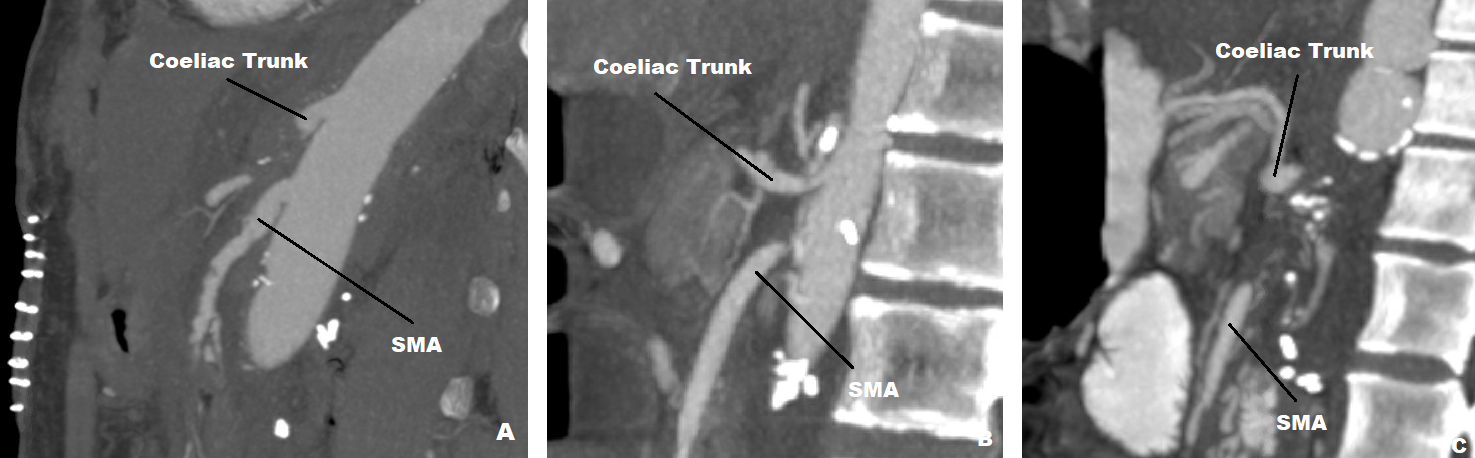
**

**Supplementary Figure 1**: Examples of visceral malperfusion. CT-angiograms of patients included in this study. A: Thrombotic occlusion of the coeliac trunk, B: Stenosis of the coeliac trunk and superior mesenteric artery, C: thrombotic occlusion of the superior mesenteric artery. SMA: superior mesenteric artery

**
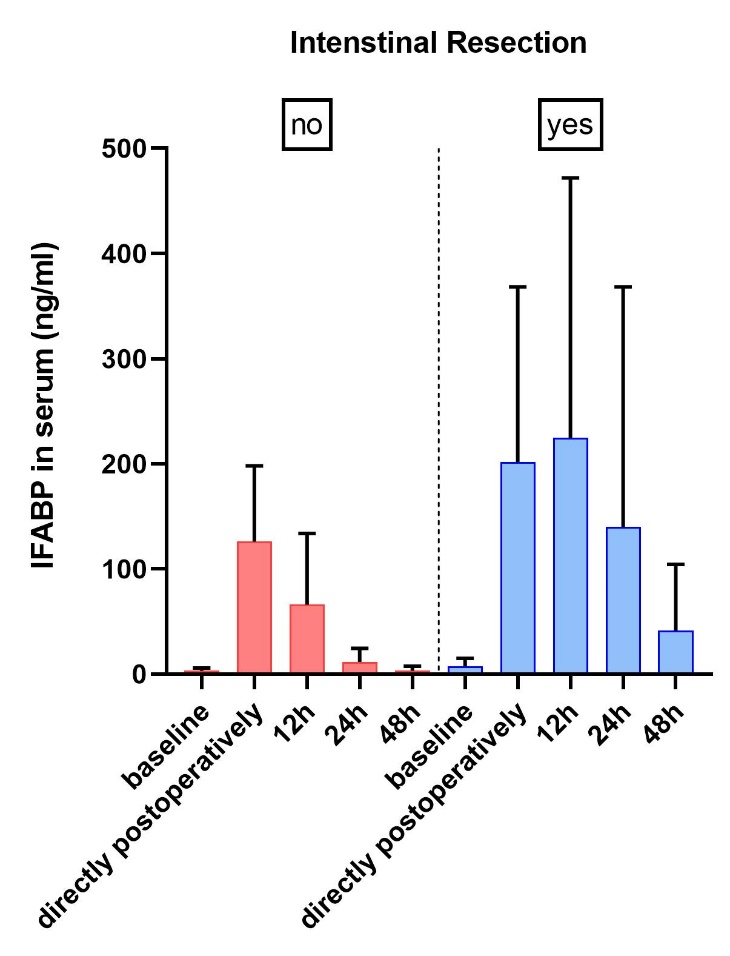
**

**Supplementary Figure 2.** IFABP levels in serum in the subgroup of patients with visceral malperfusion.
